# Remote preconditioning in normal and hypertrophic rat hearts

**DOI:** 10.1186/1749-8090-6-34

**Published:** 2011-03-23

**Authors:** Christos Voucharas, Antigoni Lazou, Filippos Triposkiadis, Nikolaos Tsilimingas

**Affiliations:** 1 Department of Cardiovascular and Thoracic Surgery, School of Medicine, University of Thessaly, Larissa 41335, Greece; 2 Laboratory of Animal Physiology, Department of Zoology, School of Biology, Aristotle University of Thessaloniki, Thessaloniki 54006, Greece; 3 Department of Cardiology, School of Medicine, University of Thessaly, Larissa 41110, Greece

## Abstract

**Background:**

The aim of our study was to investigate whether remote preconditioning (RPC) improves myocardial function after ischemia/reperfusion injury in both normal and hypertrophic isolated rat hearts. This is the first time in world literature that cardioprotection by RPC in hypertrophic myocardium is investigated.

**Methods:**

Four groups of 7 male Wistar rats each, were used: Normal control, normal preconditioned, hypertrophic control and hypertrophic preconditioned groups. Moderate cardiac hypertrophy was induced by fludrocortisone acetate and salt administration for 30 days. Remote preconditioning of the rat heart was achieved by 20 minutes transient right hind limb ischemia and 10 minutes reperfusion of the anaesthetized animal. Isolated Langendorff-perfused animal hearts were then subjected to 30 minutes of global ischemia and reperfusion for 60 minutes. Contractile function and heart rhythm were monitored. Preconditioned groups were compared to control groups.

**Results:**

Left ventricular developed pressure (LVDP) and the product LVDP × heart rate (HR) were significantly higher in the hypertrophic preconditioned group than the hypertrophic control group while left ventricular end diastolic pressure (LVEDP) and severe arrhythmia episodes did not differ. Variances between the normal heart groups were not significantly different except for the values of the LVEDP in the beginning of reperfusion.

**Conclusions:**

Remote preconditioning seems to protect myocardial contractile function in hypertrophic myocardium, while it has no beneficial effect in normal myocardium.

## Background

The heart can be protected from an episode of acute lethal ischemia/reperfusion injury by applying brief non-lethal episodes of ischemia and reperfusion either to the heart itself (ischemic preconditioning = IP) or to an organ or tissue that is remote from the heart (remote preconditioning = RPC) [1-3].

Initial enthusiasm for the beneficial effects of ischemic preconditioning of the heart in animal or human studies has given place to skepticism, since there has not yet been broad application of the method in clinical practice [4]. Controversy still exists about the efficacy of the RPC in normal hearts, as well as about the value of IP in the hypertrophic myocardium [5-10].

Larger multicenter trials would be required to confirm the results and novel methods should be employed to accurately estimate the influence of ischemic preconditioning in cardioprotection.

Moreover, remote preconditioning of the hypertrophic heart has never been studied before. Moderate cardiac hypertrophy is a common state of many physiological and pathological conditions in humans: exercise, pregnancy, hypertension, heart valve disease or myocardial infarction. We have to note that RPC may refer to the same organ and to a distant organ or tissue. Remote preconditioning of the heart regarding transient ischemia caused to another organ or tissue far from the heart, has advantage over classic ischemic preconditioning or RPC regarding transient ischemia of a region of the heart other than the region examined for sustained ischemia, since it does not compromise the myocardium [11,12].

This study was designed to investigate if remote preconditioning at a distant organ improves myocardial function after ischemia/reperfusion injury in normal rat hearts and - for the first time in world literature - to examine the action of RPC in hypertrophic rat myocardium.

## Methods

### Animals

Twenty eight male Wistar rats were used for this study. They were randomly divided into 4 groups of 7 animals each to form normal control (NC = non hypertrophic myocardium, non preconditioned), normal preconditioned (NP), hypertrophic control (HC) and hypertrophic preconditioned (HP) group.

All animals were treated according to the Guidelines for the Care and Use of Laboratory Animals stated in the Greek law (160/1991) based on European Union regulations (European Commission Directive 86/609/EEC). Furthermore, the experimental protocol was approved by our Institutional Ethical Committee.

### Model of hypertrophy

Hypertensive myocardial hypertrophy was established to 14 animals (originally 2-months of age and weighing 150-200 grams) by concurrent administration of a synthetic mineralocorticoid (fludrocortisone acetate, Institute for Pharmacological Research and Technology, IFET, Pallini Attikis, Greece) and saline for 30 days [13,14]. Corticoid/salt model of hypertrophy is a pressure overload induced cardiac hypertrophy model. In this model, hypertrophy is both concentric and eccentric, similarly to hypertrophy in humans [15]. Fourteen more two-month-old male rats were fed a normal diet for 30 days. At 3 months of age all the animals (both normal and hypertrophic heart rats) were weighing 200-250 grams and they were ready to undergo the experiment. Solid alimentation supply was the same for all animals.

The animals intended for myocardial hypertrophy were given 12.5 cc of a salt solution with fludrocortisone acetate (0.9% NaCl, 0.2% KCl, 2.54 mEq % Mg^++^, 0.002 mg % fludrocortisone acetate) to drink in place of water for the first half of every day and a free quantity of a salt solution (0.9% NaCl, 0.2% KCl, 2.54 mEq % Mg^++^) for the rest of the day, in order to ensure a standard corticoid intake of 0.00025 mg per animal per day. Per os corticoid administration was adopted instead of subcutaneous injection [10] in order to avoid additional anxiety and stress to animals because of the injection.

The heart weight to body weight ratio was used as an index of myocardial mass.

### Experiment protocol

Animals were anaesthetized by intraperitoneal injection of sodium pentothione (100 mg/kg). Heparin was delivered intravenously (300 IU/kg) through the femoral vein. The right common femoral artery (just below the inguinal ligament) of the animals that were scheduled to receive remote preconditioning was exposed and temporarily occluded for 20 minutes. Occlusion was achieved by a silicon loop tightened by a tourniquet. Then, circulation to the hind limb was restored for 10 minutes.

Next steps of the procedure were common for all the groups. The hearts were rapidly excised and placed immediately in ice-cold perfusion buffer (0°C) before being mounted on a Langendorff apparatus. The ischemic time between excision and mounting was less than 1 min. The pericardium, the pleural cavities and the peritoneal cavity were at the same time explored for effusions; the liver weights as well as the lung weights of the rats were measured in order to calculate liver weight/body weight (LiW/BW) as well as lung weight/body weight (LW/BW) ratio; the aim was to investigate heart failure. Hearts were retrogradely perfused in an isovolumetric Langendorff mode at a constant hydrostatic pressure of 100 cm H_2_O during the entire duration of the experiment. The perfusion medium was a non-recirculating oxygenated (95% O_2_, 5% CO_2_) normothermic (37°C) Krebs-Henseleit bicarbonate (KHB) buffer. KHB buffer had the following ion concentrations in mmol/L: 25 NaHCO_3_, 4.7 KCl, 118.5 NaCl, 1.2 MgSO_4_, 1.2 KH_2 _PO_4_, 2.5 CaCl_2 _and 10 glucose (pH 7.4). The perfusion apparatus was water-jacketed to maintain a constant perfusion temperature of 37°C.

To determine left ventricular pressure, a catheter with a latex balloon on its tip was inserted into the left ventricle through an incision in the left atrial appendage. The balloon was tied securely into place and filled with water to give an end diastolic pressure between 6 and 10 mmHg. The adjusted volume remained constant throughout the experiment. This allowed continuous measurement of left ventricular pressures and recording of their alterations on a fixed preload. The balloon was connected to a pressure transducer via water-filled polyethylene tubing. Three stainless steel electrodes were inserted into the epicardium of both of the atria and of the right ventricle for three leads bipolar electrocardiogram recording. Left ventricular pressure and heart rhythm were monitored continuously and recorded on a computer. All hearts were allowed to stabilize for 10 min after being mounted. Baseline measurements were recorded during this period. Hearts were allowed to beat spontaneously throughout the experiment. Lethal or threatening arrhythmias (like ventricular fibrillation, tachycardia or bigeminy) at the reperfusion period following the sustained ischemic insult were converted to normal rhythm by tapping the ventricle.

Left ventricular function was assessed by left ventricular developed pressure (LVDP), end diastolic pressure (LVEDP) and the product HR (heart rate) × LVDP. Developed pressure is defined as peak systolic minus end diastolic pressure. In this experimental model, LVDP represents the heart's contractile ability which is not influenced by preload and afterload.

Zero flow ischemia was induced by clamping of the arterial line. Sustained ischemia lasted 30 min for all series. The reperfusion time was 60 min. The experiment protocol is concisely presented in table 1. The measured values (baseline and then every 5^th ^minute after reperfusion) were committed to paper.

**Table 1:** Experiment protocol

Control groups			Isolated heart stabilization period 10 min →	**Sustained ischemia 30 min →**	Reperfusion period 60 min
Preconditioned groups	**Limb ischemia 20 min→**	Limb reperfusion 10 min →	Isolated heart stabilization period 10 min →	**Sustained ischemia 30 min →**	Reperfusion period 60 min

### Statistical analysis

NC group was compared to NP group and respectively HC group was opposed to HP group. Values were expressed as the mean ± SEM. Two-tailed unpaired t test was used to compare BW, HW/BW ratio, LW/BW ratio, LiW/BW ratio, arrhythmia incidents and baseline hemodynamic data. Regular (not matching) two way ANOVA was performed to test for any differences between hemodynamic values (LVDP, LVEDP, LVDPxHR) measured at various time points and examine if time point of reperfusion affected the result. Data in every separate group passed normality test and differences between SEMs in compared groups (raw data) were due to random sampling. A difference was considered statistically significant if p < 0.05.

## Results and discussion

Body weight (BW) was similar between normal and hypertrophic heart rats, but heart weight to body weight (HW/BW) ratio markedly differed (hypertrophic approximately 46% in excess) as shown in table 2. Body weight and HW/BW ratio did not differ between the compared groups - animals were equally distributed among the groups (data not presented). There was no evidence of heart failure in hypertrophic heart animals: no remarkable cavity effusions in hypertrophic groups and no significant difference in lung weight to body weight ratio as well as liver weight to body weight ratio between normal and hypertrophic heart animals (table 3).

**Table 2:** Body weight and heart weight/body weight ratio of normal and hypertrophic heart rats

	Normal heart rats n = 14	Hypertrophic heart rats n = 14	p
Body weight	219.8 ± 3.079 grams	222.9 ± 2.548 grams	0.444
Heart weight/body weight	0.004301 ± 0.0001466	0.006289 ± 0.0002148	< 0.0001*

Body weight did not differ between normal and hypertrophic heart rats, while heart weight/body weight ratio was considerably different. Values are expressed as the mean ± SEM. The asterisk (*) means statistically significant, p < 0.05.

**Table 3:** Lung weight/body weight ratio and liver weight/body weight ratio in normal heart animals and hypertrophic heart animals used for the experiment

	normal n = 14	hypertrophic n = 14	p
LW/BW ratio	5.534 ± 0.08274	5.396 ± 0.04775	0.190
LiW/BW ratio	44.13 ± 0.3586	44.02 ± 0.3085	0.8272

The organs potentially affected by heart failure had a similar growth in normal-heart and hypertrophic heart rats. BW = body weight, LW = lung weight, LiW = liver weight.

Values are expressed as the mean ± SEM.

Baseline (stabilization period) values for LVDP, LVEDP and LVDPxHR did not significantly differ when control groups were compared to preconditioned groups as shown in table 4.

**Table 4:** Baseline hemodynamics

baseline	NC group	NP group	p	HC group	HP group	p
LVDP	82.6 ± 4.23	79.6 ± 2.48	0.5521	91.9 ± 8.36	92.9 ± 6.11	0.9247
LVEDP	9.7 ± 0.74	9.0 ± 0.81	0.8319	10.0 ± 0.38	10.0 ± 0.95	1.0000
LVDPxHR	18664 ± 942	18556 ± 543	0.9225	18944 ± 2216	19918 ± 973	0.6944

Baseline hemodynamic values did not differ between the groups in comparison.

LVDP and LVEDP were measured in mmHg. Values are expressed as the mean ± SEM

**Figure 1:**
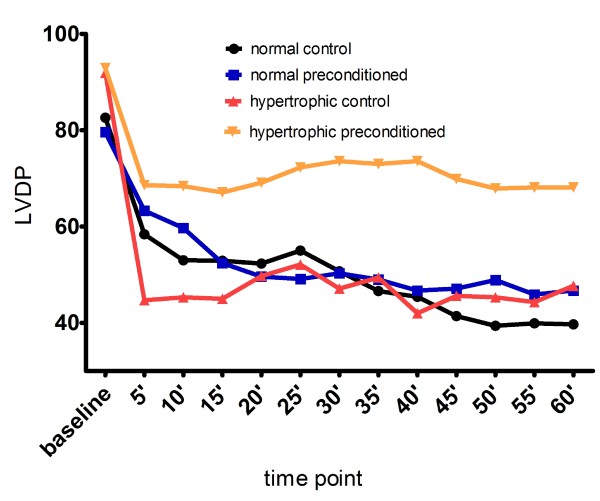
**Mean LVDP in various time points**. LVDP values in mmHg, time point in minutes.

**Figure 2:**
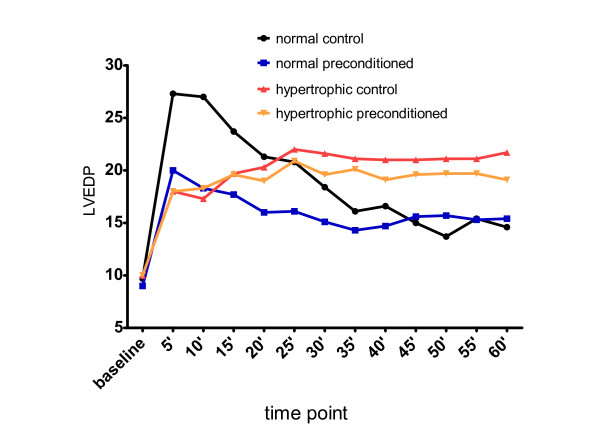
**Mean LVEDP in various time points**. LVEDP values in mmHg, time point in minutes.

**Figure 3:**
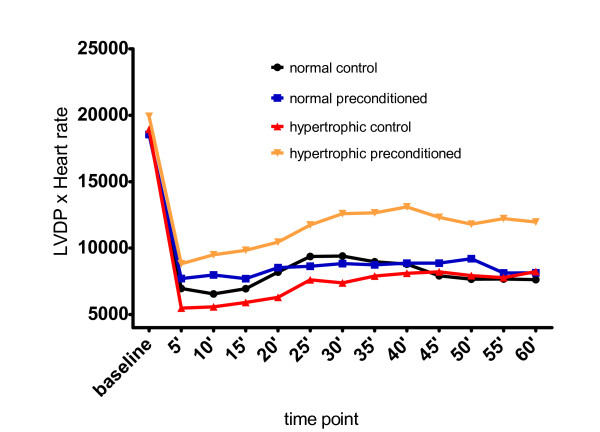
**Mean of the product LVPDxHR in various time points**. Time point in minutes.

All hearts started beating within a few seconds at the onset of reflow. Thirty-minute sustained myocardial ischemia markedly affected myocardial function during reperfusion period in all groups: LVDP was lessened, LVEDP was elevated and the product LVDPxHR was reduced in every single group and at any time point of reperfusion, in comparison to baseline measurements (figures 1, 2, 3). The differences were very significant reflecting myocardial damage after the ischemic insult (data in details not presented).

Myocardial ischemia and infarction alter not only the contractile systolic properties of the heart but also its diastolic properties. Elevation of LVEDP against fixed preload is an indication of enhanced wall stiffness of the heart. In crystalloid perfused hearts, this enhanced stiffness is attributed to the increase of myofibrillar tone and the so-called erectile or garden hose effect whose relative magnitude is dependent on the severity of myocardial damage induced by ischemia [16,17].

### Remote preconditioning influence in hemodynamics of normal (without cardiac hypertrophy) rats

**Figure 4:**
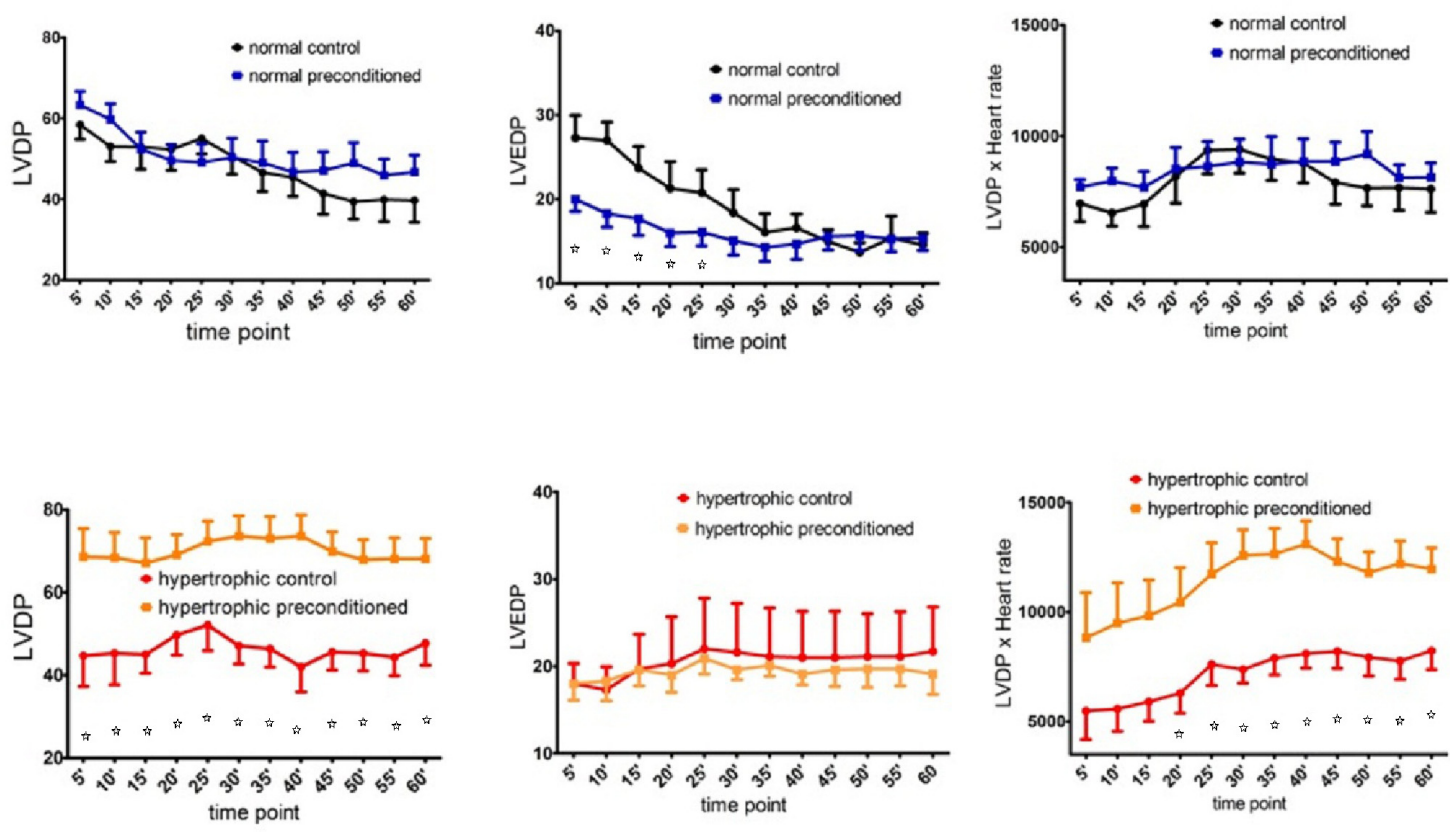
**Mean LVDP, LVEDP and LVDPxHR with SEM in every compared (control or preconditioned, normal or hypertrophic) couple of groups, in various time points**. The asterisk (*) shows significant differences. LVDP and LVEDP in mmHg, time point in minutes.

Preconditioning did not significantly affect the LVDP between the normal groups (p = 0.1314). However, time significantly influenced the values measured (p = 0.0011). As time passed, during reperfusion period, the preconditioned group retrieved from lower level; mean LVDP of the preconditioned group exceeded the normal group mean value after time point 45' (figure 4). This reflected the disproportional variation on LVEDP between the two groups in the early phase of reperfusion (figure 4). However LVDP values were comparable at whichever time point between the two groups (interaction was not significant, p = 0.9006).

LVEDP significantly differed between the two groups (p = 0.0004) and in addition time point affected the result (p < 0.0001): non preconditioned myocardium was markedly "stiffer" than preconditioned at the early phase of reperfusion; however, thirty five minutes later and till the end of reperfusion period both groups behaved in a similar way (figure 4); apparently part of myocardial damage was reversible.

Values of the product LVDPxHR were almost similar (p = 0.2464) at any time point of reperfusion (p = 0.3931) for both normal groups (figure 4). That was equally due to LVDP and HR values.

As an overall validation, hemodynamics did not vary between preconditioned and non preconditioned normal group in our investigation.

However, several animal studies have shown that brief ischemia induced in remote organs, for example, kidney, intestine, and skeletal muscle, decreased myocardial infarct size [3,11,18]. One can hypothesize that the 30 minutes of myocardial ischemia in our investigation was not long enough to cause large and permanent/irreversible damage to the heart. During the past 5 years, remote ischemic preconditioning has shown promise in small randomized controlled trials as a means of myocardial protection before paediatric and adult cardiac surgery and percutaneous coronary interventions [19]. Nevertheless, controversy still exists for both laboratory and clinical studies concerning remote preconditioning [4,5].

### Remote preconditioning influence in hemodynamics of hypertrophic heart rats

Post-ischemic LVDP was considerably higher in the preconditioned group (p < 0.0001) throughout all reperfusion time (time point influence was not significant, p = 0.9928) (figure 4). Hypertrophic control group had constantly higher LVEDP (no time affection, p = 0.9989), but difference was not significant (p = 0.4666) (figure 4).

The product LVDPxHR was markedly higher in preconditioned group (p < 0.0001) owned especially to LVDP factor (HR was almost identical between the groups - data not presented). However, for the first 15 min of reperfusion difference was not significant (p = 0.1969, 0.0873 and 0.0561 for time point 5', 10' and15' respectively), although the preconditioned group was superior all the time (time affection p = 0.01680) (figure 4).

In conclusion, RPC apparently improved post-ischemic left ventricular contractility of the hypertrophic heart according to this study, while it did not affect diastolic dysfunction.

In laboratory studies, pressure overload induced cardiac hypertrophy has been shown to be associated with a greater susceptibility to ischemic/reperfusion injury in comparison to normal hearts. A number of morphologic, metabolic, and physiologic adaptive changes in the hypertrophic myocardium contribute to this phenomenon (subendocardial underperfusion, increased membrane damage, recruitment of anaerobic glycolysis, coronary vascular turgor effect) [20-23]. This makes effort to improve hypertrophic heart resistance to ischemia/reperfusion by remote preconditioning more challenging. Our study showed a remarkable result: there was a positive effect of the remote preconditioning in the hypertrophic heart as opposed to the normal heart. It seems that the stimulus of the remote organ ischemia/reperfusion was not powerful enough to protect the normal myocardium from sustained ischemia; however the stimulus was sufficient to shield the more susceptible to ischemia hypertrophic myocardium.

Ischemic preconditioning of hypertrophic myocardium has not been studied as extensively as preconditioning of normal myocardium. Unlikeness among the results of reported investigations might be due to different protocols [8-10,24-28].

### Ventricular arrhythmia

Episodes of ventricular arrhythmia were rare in all groups. Conversion to normal rhythm was done automatically or by tapping the ventricles. Differences between the groups being compared were only due to chance (table 5).

**Table 5:** Incidences of ventricular arrhythmia at reperfusion period

	NC group	NP group	p	HC group	HP group	p
No of arrhythmias	0.54 ± 0.29	1.14 ± 0.63	0.4039	1.57 ± 0.65	0.43 ± 0.30	0.1373

Number of arrhythmias was statistically indifferent in both the couples of compared groups. Values are expressed as the mean ± SEM

Evidence exists for a heart in failure to be prone to arrhythmia when exposed to ischemia/reperfusion, but not for a hypertrophic myocardium. Very few laboratory researches deal with remote preconditioning and arrhythmia in normal hearts, thus, forming an opinion from the literature is not safe [29,30].

## Conclusions

Several strategies of protection against ischemia/reperfusion injury by preconditioning the heart have been designed. A variety of experiment animals, duration of sustained ischemia and models of brief non-lethal ischemia (cardiac and noncardiac, preconditioning, perconditioning and postconditioning) have been tried. The final aim of all these investigations is application to mankind and prevention or restriction of ischemia/reperfusion induced myocardial damage. In contrast to classic ischemic preconditioning, remote preconditioning is an intervention that does not expose myocardium to danger. Furthermore, myocardial hypertrophy is a common clinical situation. It is important to ascertain whether RPC is expected to improve the functional recovery of the heart (normal or hypertrophic).

The contribution of this study is that remote preconditioning using transient limb ischemia as the remote stimulus can advantageously be applied to hypertrophic myocardium in rats, while it has no beneficial effect in normal hearts.

## List of abbreviations

RPC: remote preconditioning; IP: ischemic preconditioning; LVDP: left ventricular developed pressure; LVEDP: left ventricular end diastolic pressure; HR: heart rate; NC: normal control; NP: normal preconditioned; HC: hypertrophic control; HP: hypertrophic preconditioned; HW: heart weight; BW: body weight; LW: lung weight; LiW: liver weight; SEM: standard error of median; KHB: Krebs-Henseleit bicarbonate;

## Competing interests

The authors declare that they have no competing interests.

## Authors' contributions

All authors have read and approved the final manuscript.

CV: conceived of the study, performed the study design and the experiment procedures, collected and analyzed data, wrote manuscript.

AL, FT, and NT: designed study, collected and analyzed data, wrote manuscript.
